# The genome of Przewalski’s horse (*Equus ferus przewalskii*)

**DOI:** 10.1093/g3journal/jkae113

**Published:** 2024-05-28

**Authors:** Nicole Flack, Lauren Hughes, Jacob Cassens, Maya Enriquez, Samrawit Gebeyehu, Mohammed Alshagawi, Jason Hatfield, Anna Kauffman, Baylor Brown, Caitlin Klaeui, Islam F Mabrouk, Carrie Walls, Taylor Yeater, Anne Rivas, Christopher Faulk

**Affiliations:** Department of Veterinary and Biomedical Sciences, College of Veterinary Medicine, University of Minnesota, Saint Paul, MN 55108, USA; Department of Veterinary Population Medicine, College of Veterinary Medicine, University of Minnesota, Saint Paul, MN 55108, USA; Division of Environmental Health Sciences, School of Public Health, University of Minnesota, Minneapolis, MN 55455, USA; ANSC 8520 Students, University of Minnesota, Minneapolis, MN 55455, USA; Department of Animal Science, College of Food, Agricultural and Natural Resource Sciences, University of Minnesota, Saint Paul, MN 55108, USA; ANSC 8520 Students, University of Minnesota, Minneapolis, MN 55455, USA; ANSC 8520 Students, University of Minnesota, Minneapolis, MN 55455, USA; ANSC 8520 Students, University of Minnesota, Minneapolis, MN 55455, USA; ANSC 8520 Students, University of Minnesota, Minneapolis, MN 55455, USA; ANSC 8520 Students, University of Minnesota, Minneapolis, MN 55455, USA; ANSC 8520 Students, University of Minnesota, Minneapolis, MN 55455, USA; Department of Animal Science, College of Food, Agricultural and Natural Resource Sciences, University of Minnesota, Saint Paul, MN 55108, USA; ANSC 8520 Students, University of Minnesota, Minneapolis, MN 55455, USA; Minnesota Zoo, Apple Valley, MN 55124, USA; Department of Animal Science, College of Food, Agricultural and Natural Resource Sciences, University of Minnesota, Saint Paul, MN 55108, USA

**Keywords:** nanopore, horse, Przewalskii

## Abstract

The Przewalski’s horse (*Equus ferus przewalskii*) is an endangered equid native to the steppes of central Asia. After becoming extinct in the wild multiple conservation efforts convened to preserve the species, including captive breeding programs, reintroduction and monitoring systems, protected lands, and cloning. Availability of a highly contiguous reference genome is essential to support these continued efforts. We used Oxford Nanopore sequencing to produce a scaffold-level 2.5 Gb nuclear assembly and 16,002 bp mitogenome from a captive Przewalski’s mare. All assembly drafts were generated from 111 Gb of sequence from a single PromethION R10.4.1 flow cell. The mitogenome contained 37 genes in the standard mammalian configuration and was 99.63% identical to the domestic horse (*Equus caballus*). The nuclear assembly, EquPr2, contained 2,146 scaffolds with an N50 of 85.1 Mb, 43X mean depth, and BUSCO quality score of 98.92%. EquPr2 successfully improves upon the existing Przewalski’s horse reference genome (Burgud), with 25-fold fewer scaffolds, a 166-fold larger N50, and phased pseudohaplotypes. Modified basecalls revealed 79.5% DNA methylation and 2.1% hydroxymethylation globally. Allele-specific methylation analysis between pseudohaplotypes revealed 226 differentially methylated regions in known imprinted genes and loci not previously reported as imprinted. The heterozygosity rate of 0.165% matches previous estimates for the species and compares favorably to other endangered animals. This improved Przewalski’s horse assembly will serve as a valuable resource for conservation efforts and comparative genomics investigations.

## Teaser

Przewalski’s horse is an endangered wild horse native to central Asia. After becoming extinct in the wild, multiple conservation efforts were used to rescue the species, including zoo breeding programs, reintroduction into the wild, protected lands, and cloning. Flack *et al*. used a blood sample to build a representative set of genes for Przewalski’s horse. This digital resource will aid species conservation.

## Introduction

The Przewalski’s horse (*Equus ferus przewalskii*), also called the tahki, is an endangered equid native to central Asia (King *et al*. 2014) whose lineage likely diverged from the domestic horse (*Equus caballus*) tens of thousands of years ago (King 2005; Der Sarkissian *et al*. 2015; MacHugh *et al*. 2017; Gaunitz *et al*. 2018). **Equus ferus* przewalskii* has a distinct short and stocky build with dun coloring and an erect mane. These horses were initially native to the steppes of central Asia, and by the 19th century inhabited only Mongolia, Tibet, and China (Bouman 1986; Wakefield *et al*. 2002). Introgression from the domestic horse, loss of natural habitat and resources, harsh climates, and hunting contributed to a severe population bottleneck and subsequent extinction in the wild in the 1960s (Food, of the United Nations AO 1986; Wakefield *et al*. 2002). All current Przewalski’s horses are the descendants of 12 wild-caught individuals and several domesticated horses (Bouman 1986; Wakefield *et al*. 2002; Turghan *et al*. 2022).

Massive conservation efforts have focused on preserving the genetic diversity of this endangered species (Ryder and Wedemeyer 1982; Ryder 1988; Bowling *et al*. 2003; King and Gurnell 2005; Goto *et al*. 2011; Orlando 2019). Targeted captive breeding and management programs, including published studbooks since 1959, have increased the Przewalski’s horse population to over 2,000 individuals (Ryder 1988; Der Sarkissian *et al*. 2015; Turghan *et al*. 2022). Reintroduction efforts starting in the 1980s have successfully established wild herds in protected lands of Mongolia, China, and Kazakhstan (King and Gurnell 2005; Robert *et al*. 2005; Kaczensky *et al*. 2007; Xia *et al*. 2014; Jiang and Zong 2019; Bernátková *et al*. 2022; Tang *et al*. 2022; Turghan *et al*. 2022), and have improved the species’ status from critically endangered to endangered (King *et al*. 2014). Recent cloning efforts through Przewalski’s Revive & Restore Project have produced two males from a cryopreserved cell line in the San Diego Zoo Wildlife Alliance Frozen Zoo (Alliance 2022a, 2022b; Restore 2024), further bolstering captive breeding efforts.

Przewalski’s horses possess additional chromosomes (2n=66) when compared with the domestic horse (2n=64) (Benirschke *et al*. 1965; Delhanty *et al*. 1979; Myka *et al*. 2003; Lau *et al*. 2008); this chromosome difference is thought to be derived from a Robertsonian translocation event (Ahrens and Stranzinger 2005; Huang *et al*. 2014). Przewalski’s and domestic horse crosses produce viable, fertile offspring with odd numbered chromosomes (n=65), in contrast to the infertile offspring of domestic horse and donkey (Equus asinus) crosses (Lippold *et al*. 2011). Investigation of the differences between domestic and Przewalski’s horse chromosome structures may facilitate improved understanding of chromosome fusion.

Recent technological advances have increased accessibility and reduced costs for generating high-quality whole-genome sequencing data (Wang *et al*. 2021). Long-read Oxford Nanopore sequencing provides genomic and epigenomic data simultaneously without amplification or bisulfite conversion (Arakawa 2023). Two major limitations of the technology, high per-base error rates and homopolymer indels, have been addressed with improved basecalling models, self-correction algorithms, and adequate depth of coverage (Delahaye and Nicolas 2021). In addition to direct capture of epigenetic base modifications, pseudohaplotype phasing permits heterozygosity estimation and allele-specific DNA methylation analysis; strict allele-specific methylation is a signature of genomic imprinting, where genes are monoallelically expressed based on parent of origin (Bajrami and Spiroski 2016; Elhamamsy 2017). The epigenome is also relevant to species evolutionary biology due to the increased mutation rate of methylated cytosines via deamination (Jaenisch and Bird 2003; Xia *et al*. 2012; Skvortsova *et al*. 2018; Yamaguchi 2019; Chang and Lu 2020; Xu *et al*. 2022).

Here, we highlight the exclusive use of Oxford Nanopore sequencing reads to provide a high-quality, highly contiguous diploid nuclear genome assembly, updated mitogenome, and DNA methylation analysis for the endangered Przewalski’s horse.

## Materials and methods

### Sample collection

**Fig. 1.**
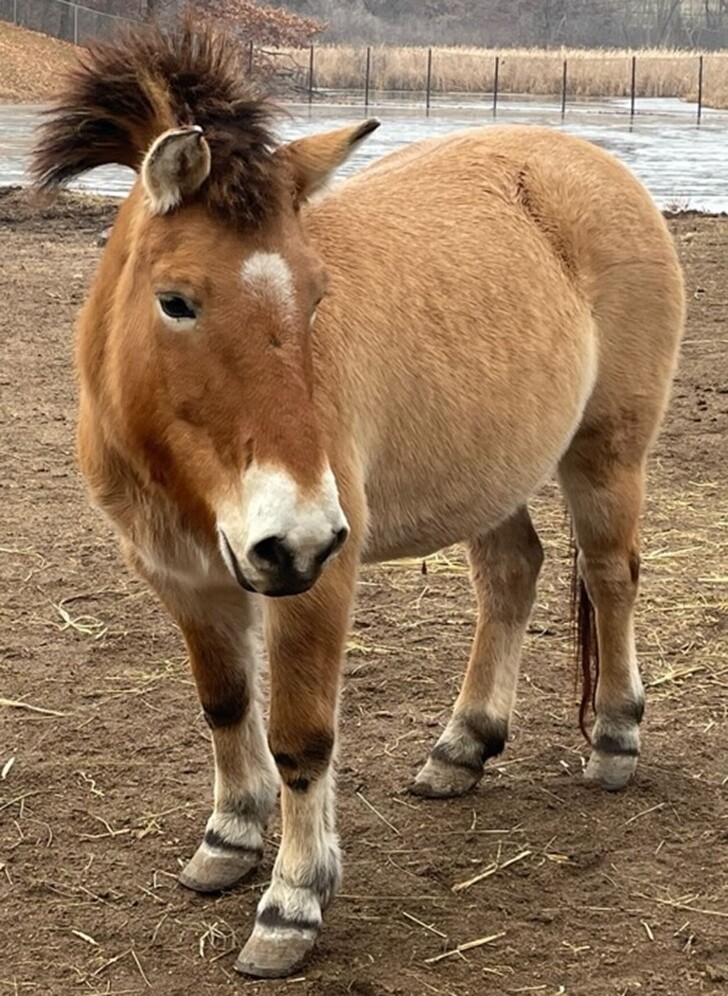
*Equus ferus przewalskii* specimen photo depicting Varuschka, the 10-year-old captive-bred mare sampled for genome assembly. Image courtesy of the Minnesota Zoo.

Varuschka, a 10-year-old captive-bred Przewalski’s mare residing at the Minnesota Zoo (Apple Valley, MN, USA; Fig. 1), was chosen for sampling. The mare was subject to routine veterinary care under anesthesia, during which 10 ml of whole blood was collected by zoo veterinarians. The Minnesota Zoo has been active in Przewalski’s horse breeding and management, with over 50 foals born since the 1970s, and contributed a stallion to reintroduction efforts in Mongolia’s Hustai National Park (Lessard 2008; Mallinger *et al*. 2023).

### DNA extraction and sequencing

Genomic DNA was extracted from blood using a MagAttract Blood DNA/RNA Kit (Qiagen, Venlo, Netherlands) according to manufacturer’s instructions. Sequencing was performed on a P2 Solo instrument (Oxford Nanopore Technologies, Oxford, UK) using a single PromethION R10.4.1 flow cell. We created two libraries using Ligation Sequencing Kit V14 (ONT catalog number SEQ-LSK-114) for native DNA sequencing. For the first library, 3 μg of DNA in 100 μl elution buffer was sheared by passage through a 28 gauge needle 30 times, library prepped, then split into three aliquots of 15 μl each. For the second library, unsheared DNA was eluted and prepped as a single 15 μl library. The first aliquot of the sheared library was loaded onto the flowcell and run for 24 hours, after which the flowcell was washed using the manufacturer’s wash kit. The remaining two sheared aliquots and the unsheared library were loaded and sequenced for the same duration on days 2, 3, and 4, respectively, with flowcell washes performed before each reload. Data were collected using 5 kHz MinKNOW version 23.07.12 (Oxford Nanopore Technologies, Oxford, UK).

Raw data from all runs were basecalled together using Dorado v0.4.3 (https://github.com/nanoporetech/dorado) with “super accuracy”model dna_r10.4.1_e8.2_400bps_supv4.2.0. Base modifications were called simultaneously using the Dorado flag --modified-bases 5mC_5hmC. Read quality was assessed using the Nanoq package (https://github.com/esteinig/nanoq).

### Genome assembly

Detailed computational methods are available in Supplementary File S2. The genome was de novo assembled using Flye v2.9 (Kolmogorov *et al*. 2019), followed by polishing using Medaka v1.6.0 (https://github.com/nanoporetech/medaka). Duplicate contigs were removed using Purge Dups v1.2.6 (https://github.com/dfguan/purge_dups). Additional manual curation was performed to remove contigs with less than 15× or greater than 500× mean coverage. One contig representing the mitochondrial genome was also removed at this stage. The resulting draft assembly was scaffolded onto the reference domestic horse genome, EquCab3.0 (GCF_002863925.1), using the RagTag package v2.1.0 (Alonge *et al*. 2022). Gaps were closed using TGS-GapCloser v1.2.1 (Xu *et al*. 2020).

Scaffolds for *E. ferus przewalskii* were assigned chromosome names based on homology to *E. caballus* synteny from EquCab3.0 as demonstrated previously (Ahrens and Stranzinger 2005). The homologous *E. f. przewalskii* scaffold to *E. caballus* chromosome 5 was split on its scaffold gaps with AWK, and the largest blocks syntenic to the *p*- and *q*-arms were labeled with their chromosome names (Supplementary File S1). The remaining smaller contigs syntenic to EquCab3.0 chromosome 5 were labeled as ChrUns in the scaffolded assembly. The FCS-adapter tool from the NCBI Foreign Contamination Screening program suite was used to detect and remove adapter and vector contamination from the final haplotype assemblies (https://github.com/ncbi/fcs).

The quality of all draft assemblies was evaluated by detecting Benchmarking Universal Single-Copy Orthologs (BUSCOs) within the *Cetartiodactyla* lineage (Manni *et al*. 2021). The reference dataset used for searching, “cetartiodactyla_odb10”, contained 13,335 genes shared by 22 species. We used the Compleasm package to calculate BUSCO scores as it is a faster, more accurate implementation of the package (Huang and Li 2023). We combined Compleasm’s single and duplicate BUSCO counts to provide a direct comparison to the standard BUSCO program’s ‘complete’ value. On average, Compleasm detected 3% more BUSCOs than the BUSCO tool v3.4.7 (Supplementary File S1). We calculated assembly N50, L50, and other statistics using the packages Assembly-Stats (https://github.com/sanger-pathogens/assembly-stats) and Quality Assessment Tool for Genome Assemblies (QUAST v5.2.0) (Mikheenko *et al*. 2018), with EquCab3.0 serving as the reference for the latter. Assembly statistics were compared with other publicly available equid genomes including the preexisting Przewalski’s horse (Burgud), domestic horse (EquCab3.0), and plains zebra (*Equus quagga*; UCLA_HA_Equagga_1.0) references.

### Repeats

RepeatMasker v4.1.4 (https://www.repeatmasker.org/) was used to identify repetitive sequences with the complete Dfam library v3.6 (https://www.dfam.org/home) as described previously (Flynn *et al*. 2020; Storer *et al*. 2021). For consistency of comparison, Repeatmasker was also run locally on the existing equid reference genomes with the same parameters.

### Variant calling and diploidization

Clair3 v1.05 (Zheng *et al*. 2022) was run to determine the number and type of variants. Heterozygosity was calculated by counting the number of variants divided by the total genome size. Variants were phased with Whatshap (Martin *et al*. 2016) and haplotagged with Longphase (Lin *et al*. 2022). BCFTools was used to swap out the phased variants in the primary assembly to generate the secondary pseudohaplotype assembly (https://github.com/samtools/bcftools).

### Gene annotation

Homology-based gene prediction was performed with Gene Model Mapper (GeMoMa, https://doi.org/10.1007/978-1-4939-9173-0_9) using EquCab3.0 transcripts as the reference. BUSCO was used in protein mode to assess gene prediction accuracy and completeness.

### Mitochondrial assembly

The mitogenome was extracted from the *E. ferus przewalskii* assembly using MitoHiFi v3.2 (Allio *et al*. 2020; Uliano-Silva *et al*. 2022). The program identifies mitogenome contigs by comparison to known mitogenomes from related species; in this case, we used the EquCab3.0 mitogenome (NC_001640.1). MitoHiFi also circularizes and annotates the putative mitogenome contig.

### Methylation

Global DNA methylation and hydroxymethylation (5 mC and 5 hmC) at cytosine-guanine dinucleotides (CpGs) were determined using modified base information stored in the initial basecalling output files (i.e. unmapped modBAMs). These files were concatenated together and aligned to the final scaffold-level assembly using Minimap2 (Li 2018, 2021). The resulting mapped modBAMs were converted to bedMethyl format using Modkit v0.2.2 (https://github.com/nanoporetech/modkit) and global 5 mC and 5 hmC percentages were calculated using AWK (https://www.gnu.org/software/gawk/manual/gawk.html#Manual-History).

For allele-specific DNA methylation, Modkit was applied to the phased modBAM generated by variant calling to generate 2 5 mC bedMethyl files separated by haplotag. Count filtering, normalization, tiling into 100 bp windows, and differential methylation testing were performed with MethylKit (Akalin *et al*. 2012; Wreczycka *et al*. 2017). Windows were deemed significantly differentially methylated regions (DMRs) if they had at least 10 CpGs, a mean absolute difference in methylation >=50%, and a Benjamini–Hochberg-adjusted *P*-value <0.05. DMRs were annotated by identifying the nearest gene with BedTools (Quinlan and Hall 2010). DMRs identified between haplotypes were visualized with MethylArtist (Cheetham *et al*. 2022).

## Results and discussion

### Assembly

We generated a total of 111 Gb of DNA sequencing data for Przewalski’s horse with a read N50 of 10,829 bp and mean quality of Q18.49. The quality of each draft assembly was assessed with parameters including N50 (i.e. length of the shortest contig at 50% of the total assembly length), L50 (i.e. smallest number of contigs whose length sum to 50% of the total assembly length), and the count of benchmark universal single-copy ortholog (BUSCO) genes. Our initial Flye run yielded a 2.59 Gb draft assembly with 6,808 contigs and an N50 of 13.6 Mb (Table 1). With an L50 of 55, the majority of the genome was assembled into relatively few contigs representing large portions of chromosomes.

**Table 1.** Contiguity and quality statistics for draft *E. ferus przewalskii* assemblies, final EquPr2 assemblies, and existing equid reference genomes.

Draft	Size (Gb)	Contigs/ Scaffolds	N50 (Mb)	L50	BUSCO complete	BUSCO single	BUSCO duplicate	Gaps (n)
Przewalski’s horse (Burgud)	2.396	53,097	0.514	1,223	89.88%	89.51%	0.37%	80,963
Domestic horse (EquCab3.0)	2.507	4,700	87.231	12	98.92%	97.74%	1.18%	6,294
Zebra (UCLA_HA-_Equagga-_1.0)	2.501	12,506	122.556	9	98.47%	95.13%	3.34%	14,637
Initial assembly (Flye)	2.594	6,808	13.697	55	98.84%	98.17%	0.67%	0
Polished (Medaka)	2.594	6,808	13.696	55	98.85%	98.19%	0.66%	0
Purged (Purge_dups)	2.503	3,411	13.974	52	98.77%	98.22%	0.55%	0
Curated	2.498	2,796	13.976	52	98.77%	98.22%	0.55%	0
EquPr2 HP1	2.500	2,146	85.15	12	98.92%	98.37%	0.55%	306
EquPr2 HP2	2.499	2,146	85.13	12	98.92%	98.35%	0.57%	306

BUSCO scores were calculated with the Compleasm tool (Huang and Li 2023). HP: pseudohaplotype.

The initial assembly was polished, purged of duplicates, and manually curated to remove contigs with coverage below 15× and 500× as they are unlikely to represent single-copy nuclear regions (Flack *et al*. 2023). This procedure resulted in the removal of 4,012 contigs and 96 Mb of sequence. Polishing with Medaka increased the complete BUSCO score by reducing the percentage of duplicate BUSCOs by 0.01%. In our initial draft, the duplicate BUSCO count was already low at less than 1% of the total; haplotig purging reduced the number of duplicate BUSCOs by 0.11% and increased N50 from 13.70 Mb to 13.97 Mb.

**Fig. 2.**
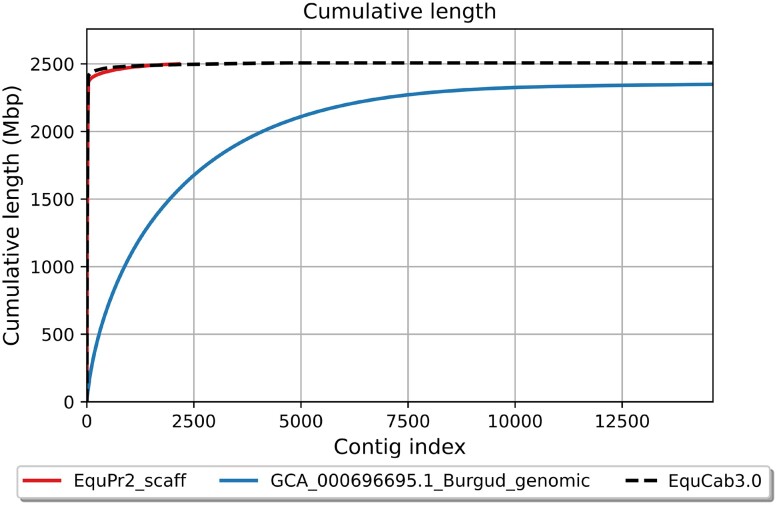
Cumulative length of EquPr2 scaffolds compared with the existing reference genomes for Przewalski’s horse (Burgud) and domestic horse (EquCab3.0).

We scaffolded the curated assembly onto the domestic horse genome (EquCab3.0); gaps were filled with reads placed by TGS-GapCloser where spanning reads could be identified. This final assembly, EquPr2, was 2.50 Gb in length with a chromosome-level N50 of 85.1 Mb and L50 of 12. EquPr2 contained 306 gaps spanning 2.03 Mb, a 5-fold and 11-fold reduction in gap length compared with EquCab3.0 and Burgud, respectively (Table 1). The contiguity of EquPr2 also matches EquCab3.0’s L50 of 12, in contrast to Burgud’s L50 of 1,223 (Fig. 2).

Our Przewalski’s horse genome had a BUSCO completeness score of 98.92%, improving from 89.88% in the previous Przewalski’s horse assembly with 25-fold fewer scaffolds and a 166-fold increase in N50. It is important to deconstruct the complete BUSCO score reported by most new genome assemblies into its component parts of single and duplicate copy percentages. Examining only the completeness score obscures the presence of haplotig missassemblies represented by high duplicate counts. For instance, the reference horse genome, EquCab3.0, is a high-quality, highly contiguous assembly with a complete BUSCO score of 98.92%, the same as our Przewalski’s horse assembly EquPr2. However, EquCab3.0 has a duplicate rate of 1.18% versus EquPr2’s duplicate rate of 0.55%. Similarly, the plains zebra genome, UCLA_HA_Equagga1.0, is even more contiguous with an L50 of 9 but has a 3.34% BUSCO duplication rate. While these differences are relatively small, duplication misassemblies have contributed to erroneous gene gain and gene family expansion findings in high-heterozygosity vertebrate genomes (Ko *et al*. 2022).

The Przewalski’s horse has 33 sets of chromosomes versus the domestic horse’s 32 due to a Robertsonian translocation where *E. caballus* chromosome 5 is homologous to *E. f. przewalskii* chromosomes 23 and 24 (Ahrens and Stranzinger 2005). Other EquPr2 chromosomes were named based on homology to domestic horse consistent with previous karyotyping (Richer *et al*. 2008; Musilova *et al*. 2007). We manually split the EquPr2 scaffold homologous to *E. caballus* chromosome 5 at every gap from the contig-level assembly and named the largest contigs as chromosomes 23 and 24 based on homology to the *p* and *q* arms of its species ortholog. Due to lack of positional certainty, the remaining 12 contigs (34.4 Mb total length) mapping to EquCab3.0’s chromosome 5 were named as chromosome unknown (ChrUn).

### Repetitive DNA

We used RepeatMasker to detect repetitive element content within the new assembly and compared it to the previous assembly, Burgud, and the domestic horse (Table 2). Given the genetic similarity of Przewalski’s horse to the domestic horse, global transposon content is nearly identical across all categories. Identification of single repeat insertions facilitated by a more contiguous Przewalski’s horse reference may be valuable for future comparative genomics investigations of these two closely related species.

**Table 2.** Repetitive DNA content for EquPr2, the existing Przewalski’s horse reference (Burgud), and the domestic horse reference (EquCab3.0).

	EquPr2 (%)	Burgud (%)	EquCab3.0 (%)
**Retroelements**	30.89	31.66	32.21
SINEs:	3.55	3.70	3.66
LINEs:	21.35	21.65	22.27
↪ L2/CR1/Rex	5.26	5.47	5.43
↪ L1/CIN4	15.88	15.96	16.63
LTRs:	6.00	6.32	6.28
↪ Retroviral	5.57	5.88	5.84
**DNA transposons**	3.44	3.62	3.56
hobo-activator	2.6	2.74	2.69
**Total repeats**	34.36	35.32	35.8

Only RepeatMasker categories reaching >1% genomic content are shown; smaller families are collapsed into the relevant parent category.

### Heterozygosity

Given the extreme population bottleneck that occurred during the near-extinction of Przewalski’s horse, it is critical to understand the genetic diversity remaining for captive breeding efforts. To this end and due to a lack of samples from Varuschka’s sire and dam, we called and phased EquPr2 variants to build an alternate pseudohaplotype assembly. Variant calling with Clair3 found 4,114,297 variants, the majority of which were single-nucleotide variants. Heterozygosity was estimated to be 0.165. This level of genetic diversity is concordant with previous microarray data from 9 Przewalski’s horses where average heterozygosity was 0.168, the highest estimate among nine members of *Equidae* excluding the domestic horse (McCue *et al*. 2012).

### Gene annotation

We detected 21,552 putative genes in EquPr2 with the ab initio gene prediction tool Gene Model Mapper (GeMoMa) (Keilwagen *et al*. 2016, 2018) using the EquCab3.0 annotation (GCF_002863925.1) as reference. The resulting protein BUSCO score was 85.6% complete (84.6% single copy and 1.0% duplicates); this score appears to be near GeMoMa’s maximum performance based on previous demonstration of similar results for existing high-quality reference genomes (Flack *et al*. 2023). The EquPr2 annotation would likely be significantly improved by the application of NCBI’s Eukaryotic Genome Annotation Pipeline (Thibaud-Nissen *et al*. 2016), which requires the availability of RNA-seq data.

### Mitochondrial genome

The mitochondrial genome was extracted from the assembly and characterized with MitoHiFi, a tool designed for long-read mitochondrial contig identification and annotation (Uliano-Silva *et al*. 2022). The resulting mitogenome for EquPr2 (CM075423.1) was 16,602 bp in length, had 99.63% sequence identity to *E. caballus* isolate TN9488, and contained 37 genes in the standard mammalian configuration (Supplementary File S1). This sequence was 58 bp shorter than the domestic horse reference mitogenome (NC_001640.1; 16,660 bp) and 10 bp longer than the existing Przewalski’s horse reference mitogenome (NC_024030.1; 16,592 bp). Visualization with NCBI’s Multiple Sequence Alignment Viewer v1.25.0 (https://www.ncbi.nlm.nih.gov/tools/msaviewer/) indicated that the shorter length was due in part to a tandem repeat block (motif ‘CACCTGTG’) in the mitochondrial control region (Supplementary Figure S1). The repeat copy number was 28 in EquCab3.0, 22 in EquPr2, and 20 in the reference Przewalski’s horse, Burgud. The EquPr2 mitogenome being 10 bp shorter than Burgud’s stemmed from short insertions and deletions (1–6 bp) throughout the sequence in addition to the smaller difference in tandem repeat copy number. Delineating misassemblies from true variation in this highly variable region of the equine mitogenome will be valuable for future comparative analyzes.

### DNA methylation

**Fig. 3.**
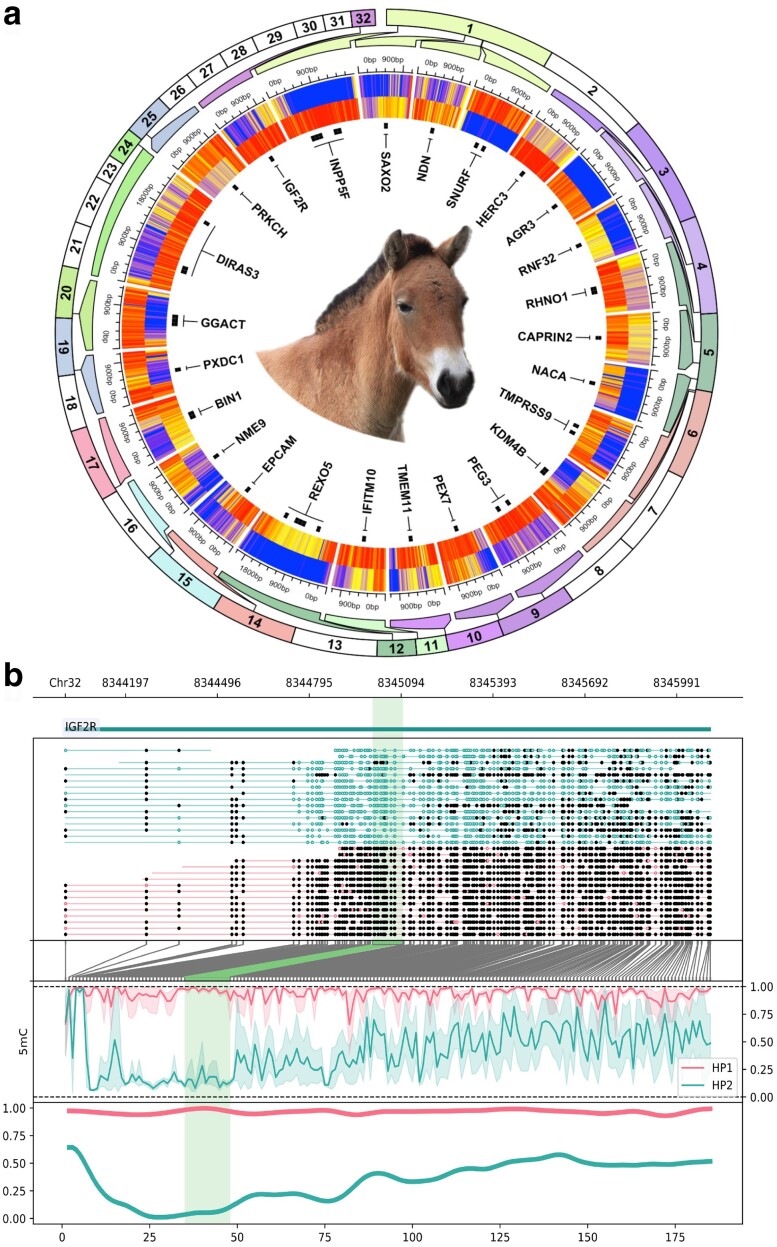
Genome-wide allele-specific DNA methylation analysis for EquPr2. a) Circular heatmap of 115 DMRs identified between EquPr2 pseudohaplotypes and labeled by nearest gene symbol. Regions are zoomed from their chromosomal position as indicated by outer track color. Each heatmap row is a pseudohaplotype and each column is a single CpG within the DMR. Methylation values range from 0% (blue) to 100% (red). b) Phased read pileup and methylation ratios for example DMR (green highlight) overlapping the known imprinted gene *IGF2R*. The top two subpanels show gene annotations and aligned reads grouped by pseudohaplotype (black circles are methylated CpGs, open pink or teal circles are unmethylated CpGs). The middle two subpanels show translation from genomic coordinates to reduced, CpG-only coordinates (gray lines) and line plots of methylation statistics (number of methylated reads/total aligned reads; *y*-axis) for pseudohaplotype across the 2.1 kb region. HP1 (pink) = primary parental pseudohaplotype; HP2 (teal) = alternate parental pseudohaplotype. The bottom subpanel shows a Hann-smoothed version of the methylation data in the middle subpanel; coordinates on the *x*-axis correspond to the Nth CpG dinucleotide in the region.

Oxford Nanopore sequencing can natively detect base modifications including 5-methylcytosine (5 mC). This feature has been applied in previous mammalian genome assemblies to evaluate allele-specific DNA methylation and identify known and putative novel imprinted genes (Flack *et al*. 2023). Globally, we found that whole blood leukocyte DNA methylation in Przewalski’s horse was 79.5% methylated and 2.1% hydroxymethylated at CpG sites genome-wide. After filtering to include CpGs with at least 10× coverage in both pseudohaplotypes, there were 18,928,678 sites available to test for allele-specific differential methylation. Counts were tiled into 100 bp windows; windows containing at least 10 CpGs with an absolute difference in methylation >=50% and Benjamini–Hochberg-adjusted *P*-value <0.05 were deemed DMRs. With these parameters, we identified 226 DMRs between EquPr2 pseudohaplotyes with a mean absolute methylation difference of 64.1% (Fig. 3). Nearest features to DMRs included known imprinted genes (e.g. *IGF2R* Barlow *et al*. 1991; Willison 1991, *INPP5F* Choi *et al*. 2005, *PEG* Kuroiwa *et al*. 1996, and *DIRAS3* Yu *et al*. 1999) and loci not previously reported as imprinted (e.g. *DLG3*). Seventy-seven DMRs (34.1%) directly overlapped predicted genes. As a consequence of random X-chromosome inactivation in a female animal, 111 of the 226 DMRs (49.1%) were located on ChrX. Imprinted genes are unique to therian mammals (i.e. among animals), influence growth, and can be perturbed by assisted reproductive technology (Hitchins and Moore 2003; Wood and Oakey 2006; Manipalviratn *et al*. 2009; Peters 2014; Karahan *et al*. 2023), making these data valuable for future investigations of Przewalski’s horse evolution, comparative genomics, and conservation.

## Conclusions

The availability of a high-quality reference genome is imperative for improved understanding of the genetic diversity of Przewalski’s horse. The lineage of the horse sampled for this paper, Varuschka, traces back to founders from Mongolia; her dam was imported from the Cologne Zoo in Germany as a part of the Species Survival Plan, and her sire was transferred from the Smithsonian’s National Zoo and Conservation Biology Institute. Here, we provide a 2.5 Gb nanopore-only genome assembly for Przewalski’s horse with an improved BUSCO score of 98.92%, 25-fold fewer scaffolds, and a 166-fold increase in N50 compared with the existing reference genome. Modified basecalls additionally facilitated allele-specific methylation analysis; significantly DMRs included known imprinted genes and potential novel loci. This genome will aid Przewalski’s horse conservation by providing a higher quality, more contiguous foundation for captive breeding, population genomics, and other efforts.

## Supplementary Material

jkae113_Supplementary_Data

## Data Availability

Supplementary File S2 contains code used to generate the presented analyzes. The EquPr2 diploid assembly and mitogenome are available under NCBI umbrella BioProject PRJNA1073944 and accessions GCA_037783145.1 (primary pseudohaplotype) and GCA_037783155.1 (alternate pseudohaplotype). Raw reads are available from the Sequence Read Archive (SRA) accession SRR28123101. Annotated genes (GFF format), variant calls (VCF format), and predicted proteins (FASTA format) are available on GSA FigShare (https://doi.org/10.25387/g3.25248139.v1). Supplemental material available at G3 online.
