# Supplementary material for: The genome of Przewalski’s horse (Equus ferus przewalskii)
Source: G3 (Bethesda). 2024 May 28;14(8):jkae113. doi: 10.1093/g3journal/jkae113 (PMC11304947; doi:10.1093/g3journal/jkae113)
Supplement: jkae113_Supplementary_Data [file jkae113_supplementary_data.zip › Supplemental_Material_G3-2024-404925.docx]

The genome of Przewalski’s horse (*Equus ferus przewalskii*)

13 May 2024

G3-2024-404925

Figure S1 Caption

**Figure S1:** NCBI Multiple Sequence Alignment Viewer result for alignment of mitogenomes from EquPr2 (CM075423.1), EquCab3.0 (NC_001640.1), and the existing Przewalski’s horse reference, Burgud (NC_024030.1). Multiple alignment suggests that the difference in mitogenome size between these assemblies stems from varying copy numbers of a tandem repeat (‘CACCTGTG’) in the 3’ control region. Smaller insertions and deletions were also observed when comparing the two Przewalski’s horse genome (e.g., gaps near position 16,140).
