# Supplementary figures and images for: The genome of Przewalski’s horse (Equus ferus przewalskii)
Source: G3 (Bethesda). 2024 May 28;14(8):jkae113. doi: 10.1093/g3journal/jkae113 (PMC11304947; doi:10.1093/g3journal/jkae113)

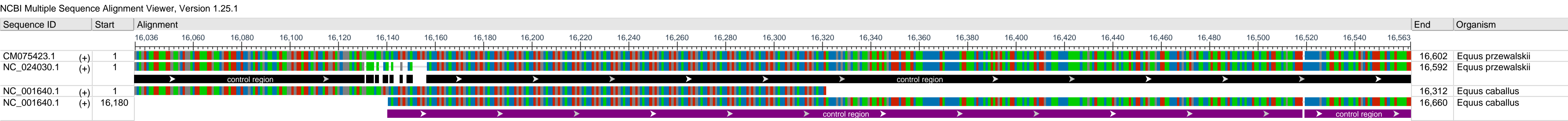

Supplement: jkae113_Supplementary_Data [file jkae113_supplementary_data.zip › Figure_S1_G3-2024-404925.pdf]
